# Self‐esteem change during the transition from university to work

**DOI:** 10.1111/jopy.12519

**Published:** 2019-10-17

**Authors:** Anne K. Reitz, Patrick E. Shrout, Jaap J. A. Denissen, Michael Dufner, Niall Bolger

**Affiliations:** ^1^ Department of Psychology New York University New York NY USA; ^2^ Department of Psychology Columbia University New York NY USA; ^3^ Department of Developmental Psychology Tilburg University Tilburg Netherlands; ^4^ Department of Psychology Universität Leipzig Leipzig Germany

**Keywords:** life transitions, self‐esteem development, the transition from university to work, young adulthood

## Abstract

**Objective:**

The current study examined whether the transition from university to work, a major developmental milestone in young adulthood, was related to stability and change in self‐esteem.

**Method:**

Self‐esteem was assessed in the last year of their master's program (T1) of 163 27‐year old students and 14 months later, when they had graduated and half of them had started a full‐time job (T2). Daily diaries were used to assess the occurrence of achievement‐ and affiliation‐related experiences on 14 consecutive days at T1 and T2. We compared the full‐time job beginners and a comparison group without a full‐time job with regard to their mean‐level change, rank‐order stability and correlated change of self‐esteem and daily experiences.

**Results:**

First, job beginners increased in self‐esteem, but the difference to the mean‐level change of the comparison group was only small. Second, self‐esteem was less stable among job beginners than among the comparison group. Third, the changes in achievement‐related daily experiences and self‐esteem correlated positively in the job‐beginner group but not in the comparison group.

**Conclusions:**

The findings underline the role of daily experiences during life transitions for individual differences in self‐esteem change. The discussion calls for accounting for unique transition experiences to advance theory and research on self‐esteem development.

## 1 INTRODUCTION

Self‐esteem, a person's evaluation of his or her value, lays important groundwork for successful life span development. Self‐esteem in earlier life predicts later physical and mental health, satisfaction with relationships and work, economic prospects and longevity (Orth, Robins, & Widaman, 2012; Sowislo & Orth, 2013). Understanding the antecedents of self‐esteem development is therefore not only of great interest for researchers, but also for therapists, educators and policy makers. Life transitions might be an especially impactful antecedent of self‐evaluative traits such as self‐esteem because self‐esteem is a central indicator of one's subjective experience of success and failure in life (Crocker & Wolfe, 2001; Hogan & Roberts, 2004). Previous research has studied self‐esteem change during the final college years (Chung et al., 2014), marriage (Chen, Enright, & Tung, 2016), and parenthood (Van Scheppingen, Denissen, Chung, Tambs, & Bleidorn, 2018). The transition from education to the workforce, a major developmental milestone in young adulthood, has however not yet been studied. Another major gap in the literature is the incomplete knowledge of how life transitions influence self‐esteem development. To address these gaps, the first aim of this study was to examine the impact of starting full‐time work after completing university on average change in self‐esteem. The second aim was to study the impact of starting full‐time work on individual differences in change. The third aim was to examine whether changes in self‐esteem are related to changes in the daily experiences to obtain first insights into mechanisms of self‐esteem stability and change.

### 1.1 Self‐esteem development in young adulthood

To understand self‐esteem development, it is necessary to distinguish between two types of stability and change. Mean‐level change describes the average change in same‐aged individuals of the population and thus provides insights into the normative development. Rank‐order stability quantifies the (in)stability of the relative standing of individuals over time (the more individuals differ in change, the lower rank‐order stability typically becomes; Robins, Fraley, Roberts, & Trzesniewski, 2001). In the following, we first review evidence on mean‐level change, followed by evidence on rank‐order stability in self‐esteem in young adulthood.

#### 1.1.1 Mean‐level change

Research has consistently reported average increase in self‐esteem across young adulthood that starts in late adolescence and continues to midlife (Erol & Orth, 2011; Orth et al., 2012; for a review see Orth & Robins, 2014). These findings stimulated research examining whether the high density of normative life transitions during young adulthood contributes to these mean‐level changes. There is evidence pointing toward increase in self‐esteem when being engaged in a long‐term romantic relationship as compared to individuals who do not experience it (Lehnart, Neyer, & Eccles, 2010; Luciano & Orth, 2017). With regard to parenthood, some studies showed declines (Bleidorn, Arslan, et al., 2016; Chen et al., 2016) and one study found that self‐esteem decreased during pregnancy, increased until 6 months after birth, and gradually decreased thereafter (Van Scheppingen et al., 2018). A study on the college experience found small increases from the beginning to the end of college, although there was an initial drop (Chung et al., 2014; see also Shim, Ryan, & Cassady, 2012), which is in contrast to a study reporting no change (van der Velde, Feij, & Taris, 1995).

In sum, there is evidence indicating that mean‐level changes in self‐esteem are related to life transitions in the domains of romantic relationships, parenthood, and education. Interestingly, effects of life transitions on mean‐level change were mixed: most often they were positive, but sometimes they were negative or absent. Evidence for the transition to work is yet missing.

#### 1.1.2 Rank‐order stability

If rank‐order stability during a life transition is low, individuals who had a relatively high level in self‐esteem within a sample can have a low relative standing at a later time point and vice versa, irrespective of any general trend. Such a change pattern would be concealed by solely focusing on mean‐level change. It is therefore important to describe not only mean‐level change, but also rank‐order (in)stability as an indication of whether a transition impacts the extent of inter‐individual differences in change (Robins et al., 2001). Knowledge of individual variability in change is also needed to understand change processes: only when we know to what extent individuals differ in their self‐esteem change can we examine the reasons for this variability.

Research on normative rank‐order stability in young adulthood has reported moderate to high stability (Donnellan, Kenny, Trzesniewski, Lucas, & Conger, 2012; Erol & Orth, 2011; Trzesniewski, Donnellan, & Robins, 2003). A recent large‐scale study reported coefficients around .9 in three‐year retest intervals for the age group 20 to 29 (Kuster & Orth, 2013), which is comparable to the stability found for the Big Five traits (Roberts & DelVecchio, 2000). These findings suggest that self‐esteem is trait‐like and that changes tend to be slow and build up gradually over long periods of time. In a recent review article, Orth and Robins (2014, p. 4) conclude from these findings that “Despite theoretical claims to the contrary, self‐esteem does not fluctuate continuously over time in response to the inevitable mix of successes and failures we all experience as we go through life.”

This conclusion is based on findings from large, population‐based studies in which environmental circumstances were relatively stable. However, the stability of self‐esteem may be lower in times of environmental changes. There is some evidence for environmental changes predicting change in rank‐order stability in self‐esteem. In a study on high school students, students participating in an international exchange year had lower rank‐order stability (*r* = .68) than control students (*r* = .82; Hutteman, Nestler, Wagner, Egloff, & Back, 2015). The high rank‐order stability in self‐esteem hence seems to decrease in the presence of a major life event. These findings are in line with the notion that individuals vary in how they experience the same type of life event (Trzesniewski et al., 2003). As a result, unique reactions to life transitions might lead to individual differences in change and thus likely also to low rank‐order stability. Research on whether life transitions lead to rank‐order instability is however scarce and comparison groups are often lacking, which are needed to disentangle maturational and transitional changes.

### 1.2 Understanding self‐esteem development during life transitions

Recent evidence suggests that self‐esteem can change as a function of life events, but little is known about what evokes these changes. A growing number of researchers agree that life transitions influence trait development not directly, but indirectly through altering social circumstances in everyday life (e.g., Hogan & Roberts, 2004). The key to understanding self‐esteem development during life transitions might thus be to investigate the associated daily life experiences that change during a transition. However, empirical evidence on how life transitions impact daily experiences that drive self‐esteem change is scarce.

Furthermore, it is unclear what types of experiences explain self‐esteem change in young adulthood. James (1890) proposed that self‐esteem rises and falls in response to external factors such as successes and failures in relevant life domains. It has since however been open to debate which domains are particularly relevant for self‐esteem development. The so‐called two‐factor approach highlights two domains that are particularly relevant, related to two correlated dimensions of self‐esteem: competence and worthiness (Mruk, 2013; Tafarodi & Swann, 2001). Competence is the evaluation of oneself as a causal agent that is a source of power and efficacy, which is based on abilities and skills. Worthiness or self‐liking relates to the evaluation of one's social worth as in one's character and attractiveness. This dichotomy maps onto the two general dimensions of psychological functioning discussed by other researchers. For example, researchers have distinguished agency (competence or “work”) from communion (warmth or “love”; for a review of definitions see Paulhus & Trapnell, 2008). In the motive disposition literature, this distinction resembles achievement versus affiliation, respectively (McClelland, 1985, which we will use from now on given our experience measure).

Even though both affiliation‐ and achievement‐related experiences might be important for self‐esteem, there is disagreement about which of the two factors is more important. The most prominent theory highlighting the role of affiliation‐related experiences is sociometer theory, which understands self‐esteem as a subjective monitor of one's relational evaluation (Leary & Baumeister, 2000). Although the authors concede that achievement experiences may also be relevant for self‐esteem as long as they convey information for one's relational value, affiliation experiences are at the center of this theory as they are most indicative of one's relational value. The other line of research makes the contrasting proposition that self‐esteem is dominated by agentic information. The Double Perspective Model proposes that individuals typically assume the agentic perspective when they think about themselves, whereas communal information is relevant when thinking about others (see Wojciszke, Baryla, Parzuchowski, Szymkow, & Abele, 2011; for hierometer theory see Mahadevan, Gregg, Sedikides, & de Waal‐Andrews, 2016).

Whereas there is evidence for the role of both affiliation‐ and achievement‐related experiences for self‐esteem, a developmental framework may be necessary to identify which daily experiences are most relevant for self‐esteem change during age‐graded life transitions. That is, one necessary refinement of this debate regarding the relative importance of affiliation and achievement for self‐esteem could be that contingencies may change as the relative importance of affiliation‐ and achievement‐related experiences varies across developmental periods. Developmental task theory (Havighurst, 1972) might be useful to help identify which daily experiences are most relevant for self‐esteem development during life transitions. This theory contends that individuals differ in how well they master the transition into new life phases, which indicates their current and future developmental success.

As self‐esteem is responsive to experiences of success and failure in domains in which one has staked one's self‐worth (Crocker & Wolfe, 2001), it may rise and fall depending on how well the demands associated with the salient developmental task are mastered (cf. Hogan & Roberts, 2004; Robins, Trzesniewski, Tracy, Gosling, & Potter, 2002). Accordingly, the observed mean‐level increase in self‐esteem across young adulthood may result from the majority of young adults’ mastery of age‐graded tasks. However, focusing on the developmental transition of parenthood has not resulted in support for this possibility, however, on the contrary, the transition to parenthood has rather been associated with decrease in self‐esteem (Bleidorn, Arslan, et al., 2016; Van Scheppingen et al., 2018). A more promising possibility might therefore be to study the transition to work as a possible predictor of self‐esteem increase.

### 1.3 Self‐esteem development in the transition from university to work

The transition from education to work is a major, formative developmental milestone in young adulthood (Schoon & Silbereisen, 2009). As the pursuit of higher education and the resulting delay of entering the labor force become an increasingly normative life path (Hutteman, Hennecke, Orth, Reitz, & Specht, 2014), the transition from university into the workforce in particular deserves more attention. This transition has become increasingly challenging in recent decades due to uncertain labor markets and the expectation to not just find a job that pays the bills but also paves the way to a fulfilling career (Vuolo, Staff, & Mortimer, 2012). Work transitions pose various opportunities for experiences of success and failure that are critical for the evaluation of the self, such as new role expectations, new responsibilities, and intellectual challenges. Corresponding transitions are therefore an ideal testing ground to examine whether experiences of success in the job transition should be followed by an increase in self‐esteem, whereas experiences of failure should be followed by a decrease in self‐esteem.

Following the notion that self‐esteem is particularly responsive to mastery experiences of the salient developmental task, self‐esteem should be related to achievement‐related experiences that inform about job success. If, for instance, a student with low self‐esteem enters work life and performs well, this experience might stimulate increases in self‐esteem. If this person, however, fails to meet the expectations at work, his or her level of self‐esteem may remain low. Self‐esteem may show a mean‐level increase as most individuals on average may fulfill stable work roles and master the transition successfully. However, considering that the time after graduation is often experienced as challenging and uncertain (Perrone & Vickers, 2003), a share of the population may in fact experience a decrease. The profound changes that a work transition brings may thus lead to a reshuffling of rank‐order differences, even when mean levels remain stable.

Apart from work‐related experiences, job beginners may differ in the changes in their social relationships during the transition to work that may impact self‐esteem change (Leary & Baumeister, 2000). Job entry brings both gains and losses in young adults’ social networks. While job entry provides opportunities for young adults to broaden the network by including colleagues, they may have difficulty fulfilling social needs and maintaining existing social ties.

In sum, theory and research point to the relevance of both affiliation‐ and achievement‐related experiences for self‐esteem development. The present study aims to contribute to the question whether and to which degree achievement‐ and affiliation‐related daily experiences affect self‐esteem development during the transition to work. Based on the rationale that self‐esteem might be particularly susceptible to those experiences that inform about the mastery of a salient life transition, changes in the achievement‐related domain might be particularly impactful during a transition from university to work.

### 1.4 The present study

The goal of the present study was to examine self‐esteem stability and change during a transition from university to work. This transition provides an ideal opportunity to study self‐esteem stability and change, as it changes the structure and content of everyday life, including changes in the environmental demands that provide ample opportunities and challenges relevant for the self. The specific aims were threefold. First, we examined whether the transition from university to work was associated with mean‐level change in self‐esteem. Based on previous research, we expected an increase in self‐esteem in those who started a full‐time job after graduation, compared to those who did not. Second, we examined whether the transition from university to work was associated with individual variability in self‐esteem change as indicated by rank‐order stability. Based on the notion that life transitions involve manifold environmental changes that vary across individuals, we expected that full‐time job beginners would show less rank‐order stability than those who do not make this transition. Third, we examined whether change in self‐esteem was related to change in daily experiences. Based on the notion that self‐esteem is responsive to feelings of mastery of developmental tasks, we expected that daily experiences that indicate success in the achievement and affiliation domains would be linked to increase in self‐esteem and daily experiences that indicate failure would be linked to decrease in self‐esteem. We expected achievement‐related daily experiences to be particularly relevant for self‐esteem change based on the rationale that they are the most salient indicators of the degree of mastery of the transition to work.

To address our aims, we analyzed data from a quasi‐experimental longitudinal study of 163 German master's students who were tested over a 14‐month period as they graduated from university. Self‐esteem was assessed before students’ graduation and afterwards, when approximately half of them have started a full‐time job and half of them did not. This design is unique as it allowed for the first time to compare the self‐esteem change of university graduates who made the transition to full‐time work to those who have not (yet) made the transition to disentangle transitional from intrinsic maturational processes. The inclusion of a comparison group that does not experience the transition and the assessments before and after the transition fulfills all the essential preconditions for examining change in response to a major life transition (Specht, 2017). As we ruled out potential group differences at T1 (pre‐existing self‐esteem change and selection factors related to obtaining full‐time employment), our study approximated a natural experiment of self‐esteem change.

Another strength of the design is the assessment of the unique transitional experience by sampling daily experiences before and after graduation. We asked all participants to report daily satisfying and frustrating achievement‐ or affiliation‐related experiences on 14 consecutive days. The daily experiences tapped into typical daily positive and negative daily stress in the domains of achievement or affiliation, which theory and research considered relevant for self‐esteem. We aggregated these daily experiences for each of the two waves to obtain an indicator for the average daily experiences at T1 and T2. Daily diary reports have been shown to be less biased indicators of the individual's daily experience than retrospective assessments (Bolger, Davis, & Rafaeli, 2003). The measurement of daily experiences provided a unique opportunity to test whether daily experiences contribute to self‐esteem development and thus provide first insights into potential explanations for self‐esteem change during the transition to work.

## 2 METHODS

### 2.1 Participants

The study was part of a large‐scale longitudinal investigation on the transition to work. Participants were students at universities in or near Berlin who had registered their master's thesis and were scheduled to complete their degrees in the next 6 months. The completion of a master's degree marks the end of higher education in Germany, as graduates usually transition into the labor force. Psychology students were not allowed to participate due to potential familiarity with the measures and procedures. All other fields of study were sampled to be representative according to official records (Statistisches Bundesamt, 2011; 12% engineering, 18% natural sciences, 36% law, business, social sciences, 23% languages, cultural sciences). Participants received a compensation of 120 Euro and feedback about their personality.

Data were collected in two waves, 14 months apart (*M* = 62 weeks, *SD* = 6) in 2012/2013. At T1, 209 students participated and of these 191 also participated at T2 (retention rate = 91%). We excluded 12 participants who had not yet graduated at T2 and 16 participants who had missing data on graduation status. The final sample hence consisted of 163 participants who had graduated with a master's degree between T1 and T2. The mean age was 27.08 (*SD* = 2.84, range 22–36) at T1 and 69% was female. We found no evidence for selection bias, as those who were excluded from the study did not differ in their self‐esteem at T1 (*M* = 3.27, *SD* = 0.58) from those who were included (*M* = 3.23, *SD* = 0.53; *t*(205) = 0.45, *p* = .938, *d* = .07). We also found no differences in any of the event variables, demographic variables or other potentially relevant variables of the larger longitudinal study (see Supporting Information).

### 2.2 Study design

The study design allowed to compare self‐esteem change in individuals who experienced a transition from university into full‐time work to individuals who did not have this experience. At T2, about half of the graduates had started a full‐time job (*n* = 78), henceforth called the job‐beginner group, and half of them had not yet started a full‐time job (*n* = 85), henceforth called the comparison group. The comparison group consisted of individuals who had part‐time jobs (*n* = 34), several part‐time jobs (*n* = 18), internships (*n* = 7), or were unemployed (*n* = 26).

To qualify as a natural experiment, the two groups should differ in no characteristics other than experiencing the transition into a full‐time job or not. At T1, the groups did not significantly differ in level of self‐esteem, the daily experiences (see Table 1), gender (68% female in job‐beginners vs. 71% in comparison), or age (*M*(*SD*)=26.87 (2.57) versus 27.27 (3.07)). There were also no group differences in any other variable in the larger data set (see Supporting Information). Within the comparison group (those unemployed, in part‐time jobs, or internships), there were also no significant differences in any of the mentioned variables.

**Table 1:** Descriptive statistics and zero‐order correlations of the manifest self‐esteem and daily experience variables at all waves per group

		1	2	3	4	5	6	7	8	9	10	*M*	*SD*
1	Self‐esteem T1	−	0.51**	0.04	−0.08	−0.16	0.06	0.22*, a	0.00	−0.29*, a	−0.21	3.29	0.51
2	Self‐esteem T2	0.85**	−	0.08	0.07	−0.18	0.01	0.07	0.23*, a	−0.17	−0.33**	3.40	0.51
3	Satisfying affiliation T1	0.36**	0.40**	−	0.45**	−0.13	0.11	0.15	0.03	−0.02	0.00	3.46	0.66
4	Satisfaction affiliation T2	0.22*, a	0.30**	0.51**	−	−0.01	−0.10	0.21	0.03	−0.02	0.12	3.31	0.72
5	Frustrating affiliation T1	−0.44**	−0.33**	−0.36**	−0.27*, a	−	0.45**	0.07	0.15	0.42**	0.30**	1.98	0.32
6	Frustrating affiliation T2	−0.40**	−0.40**	−0.23*, a	−0.45**	0.48**	−	0.09	−0.01	0.27*, a	0.34**	1.92	0.38
7	Satisfying achievement T1	0.29**	0.28**	0.29**	0.20	0.06	−0.01	−	0.47**	−0.29**	0.03	2.82	0.56
8	Satisfying achievement T2	0.27*, a	0.33**	0.42**	0.14	0.12	−0.05	0.60**	−	−0.07	−0.32**	2.97	0.60
9	Frustrating achievement T1	−0.33**	−0.32**	−0.28*, a	−0.15	0.15	0.14	−0.47**	−0.39**	−	0.49**	2.85	0.60
10	Frustrating achievement T2	−0.46**	−0.46**	−0.21	−0.16	0.10	0.31**	−0.25*, a	−0.39**	0.64**	−	2.48	0.60
	*M*	3.17	3.21	3.39	3.54	2.08	2.01	2.79	2.88	2.84	2.60	−	−
	*SD*	0.55	0.50	0.59	0.69	0.38	0.43	0.64	0.62	0.64	0.71	−	−

Note

Correlations, means, and standard deviations for the comparison group are reported below the diagonal, those for the job‐beginner group are reported above the diagonal.

* *p* < .05;

** *p* < .01.

Participants had an online account that allowed them to fill in the questionnaires and to track their progress. At each wave, they completed questionnaires and a 14‐daily diary assessment.

### 2.3 Measures

#### 2.3.1 Job status

Participants completed a questionnaire at T2 in which they indicated whether they had started a job or not. The response categories were: (a) full‐time job, (b) part‐time job, (c) several part‐time jobs, (d) internship, and (e) unemployed. Participants in Category 1 were considered as the *job beginners* and those in the other categories as the *comparison group*.

#### 2.3.2 Self‐esteem

The Rosenberg Self‐Esteem Scale (Rosenberg, 1965) was administered at both waves. Participants rated their agreement to 10 statements on a 5‐point Likert scale (1 = strongly disagree to 5 = strongly agree). Cronbach's alphas for job beginners and the comparison group were .88 and .90 at Time 1 and .88 and .87 at Time 2.

#### 2.3.3 Daily experiences

Participants completed online daily diaries on 14 consecutive days at both waves. At the end of each day, participants were requested to indicate the extent to which specific experiences had occurred during the day on a scale from 1 (*completely disagree*) to 5 (*completely agree*). The event questionnaire was developed to assess a wide range of typical daily experiences based on the motive domains (or social needs) of affiliation, achievement, and power (McClelland, 1985). Half of the statements describe experiences that satisfy these motives and half of the experiences describe experiences that frustrate these motives. Based on pilot testing, daily experiences were selected that occurred on average neither too frequently, nor too infrequently. As shown in Table 1, correlations between these aggregated experiences were low to moderate, which indicates sufficient independence between event domains.

We selected those items from these questionnaires that were relevant to our study: 10 items that measured achievement‐ and affiliation‐related experiences. Satisfying achievement experiences were “I exceeded my own expectations at work or studying” and “I improved my abilities.” Frustrating achievement experiences were “I achieved less than planned” and “I didn't succeed at work or studying.” Satisfying affiliation experiences were: “I was with people who I like,” “I talked to a close person,” “I spent a lot of time with friend, partner, family.” Frustrating affiliation experiences were: “I was alone for extended periods of time today,” “A trusted person didn't have time for me,” “I fought with a close person.”

Cronbach's alphas for the daily experiences at T1/T2 were as follows: satisfying achievement: .73/.65; frustrating achievement: .77/.72, satisfying affiliation: .80/.86; frustrating affiliation: .22/.43. Alphas were high except for the latter, which was due to the fact that the experiences were unlikely to occur on the same day (e.g., “was alone for extended periods” and “fought with a close person”) and hence alpha does not estimate reliability accurately for that scale (McNeish, 2018). All daily experiences were broad enough so that they could apply to both groups. Cronbach's alphas were comparable for the job‐beginner group and the comparison group (see Supporting Information). We aggregated the daily experiences of achievement‐ and affiliation‐related experiences per wave to capture the average daily experience at both waves.

### 2.4 Analytic strategy

#### 2.4.1 Structural equation modeling (*SEM*)

We conducted *SEM* using Mplus 7.4 (Muthén & Muthén, 1999–2015) to examine all three research aims. We used latent‐variable modeling with item‐parcels to adjust for measurement error. We aggregated the 10 self‐esteem items into three parcels using the item‐to‐construct balance parceling technique (Little, Cunningham, Shahar, & Widaman, 2002). We specified a single‐construct model with all self‐esteem items in a factor analysis. We used the ranking of the item‐to‐construct loadings as a guide to balance item discrimination and difficulty across the three parcels (i.e., the three items with the highest loadings anchored the parcels and the three items with the next highest loadings were added to the anchors in an inverted order and so forth). We did not specify daily experiences as latent variables because they were formative, not reflective indicators as their aggregation indicates the general experience of affiliation‐ and achievement‐related experiences.

We assessed model fit using the Comparative Fit Index (CFI), the Tucker–Lewis index (TLI), and the Root‐Mean‐Square Error of Approximation (RMSEA). CFI and TLI values of .90 and .95 or above and RMSEA values of .05 and .08 or below indicate acceptable and excellent fit to the data, respectively (Marsh, Hau, & Grayson, 2005). We assessed differences in model fit by using the chi‐square difference test and the CFI difference criterion (we accepted a more constrained model if the difference in CFI was less than .002; Meade, Johnson, & Braddy, 2008).

#### 2.4.2 Measurement invariance

Prior to our main analyses, we tested for measurement invariance of self‐esteem across time and groups. As the results were consistent with invariance, subsequent models were based on parsimonious time‐ and group‐invariant measurement models (see Table S3 in the Supporting Informations). Hence, mean‐level and rank‐order changes and group differences therein can be meaningfully interpreted.

#### 2.4.3 Analytic procedure

To address the first aim, we estimated mean‐level changes by testing the difference between latent self‐esteem variables at T1 and T2 (i.e., the slopes) in latent change models (McArdle & Nesselroade, 1994). We estimated the level (intercept) and change (slope) of the latent variables by having the indicators at both time points load on one latent variable (the indicators were fixed at 1 except the T1 indicators for the slopes, which were fixed at 0). The resulting change scores are latent variables that represent the error‐free difference between the scores at the two measurement occasions (Ferrer & McArdle, 2010). To address the second aim, we estimated rank‐order stability by specifying autoregressive paths between latent self‐esteem variables at T1 and T2. To address the third aim, we specified latent change models to estimate correlated change in self‐esteem and the daily experiences (cf. McArdle & Nesselroade, 1994). A positive correlation indicates that individuals who show increase in self‐esteem show concurrent increase in the experience of the daily events.

#### 2.4.4 Multiple group models

We used multiple group models to compare the job‐beginner group and the comparison group with respect to the different change indicators. To this end, we specified multiple group models that provide maximum flexibility in testing group differences in all parameters of *SEM* and thus are ideally suited to examine group differences in the measurement model and in mean‐level change, rank‐order stability, and correlated change. To test group differences in a parameter, we compared two nested *SEM* models, one with the parameter of interest constrained to be the same across the two groups and the other one without equality constraints. If the more constrained model fits significantly worse than the unconstrained model, this indicates group differences in this parameter.

## 3 RESULTS

Table 1 presents the descriptive statistics and correlations among the study variables at both waves for both groups. We estimated a sequence of multiple group models to examine group differences in mean‐level changes (Aim 1), rank‐order stability (Aim 2), and correlated changes between self‐esteem and the daily event categories (Aim 3). Table 2 shows model fit statistics and Table 3 shows the coefficients. Our syntax and the data can be found at https://osf.io/cqwxh and a preprint can be found at https://psyarxiv.com/dxkfq/.

**Table 2:** Model fit statistics and multiple group comparisons

Models	*χ^2^*	*df*	CFI	TLI	RMSEA	90% CI	ΔModel*, a	Δ*χ* ^2^	Δ*df*	*p*
1A Mean‐level change, unconstrained	35.48	32	0.995	0.995	0.037	[.000, .092]				
1B Mean‐level change, constrained	38.38	33	0.992	0.993	0.045	[.000, .096]	1A	2.90	1	.088
2A Rank‐order stability, unconstrained	34.09	29	0.993	0.992	0.046	[.000, .100]				
2B Rank‐order stability, constrained	39.94	30	0.986	0.986	0.269	[.000, .112]	2A	5.85	1	.016
3 Correlated change										
3.1A Achievement satisfying, unconstrained	46.75	47	1.000	1.000	0.000	[.000, .072]				
3.1B Achievement satisfying, constrained	53.12	48	0.993	0.992	0.036	[.000, .083]	3.1A	6.365	1	.012
3.2A Achievement frustrating, unconstrained	70.25	47	0.971	0.965	0.078	[.035, .114]				
3.2B Achievement frustrating, constrained	73.73	48	0.968	0.963	0.081	[.040, .116]	3.2A	3.479	1	.062
3.3A Affiliation satisfying, unconstrained	43.66	47	1.000	1.005	0.000	[.000, .064]				
3.3B Affiliation satisfying, constrained	43.83	48	1.000	1.007	0.000	[.000, .062]	3.3A	0.170	1	.680
3.4A Affiliation frustrating, unconstrained	57.39	47	0.986	0.984	0.052	[.000, .094]				
3.4B Affiliation frustrating, constrained	57.99	48	0.987	0.985	0.051	[.000, .092]	3.4A	0.600	1	.439

Abbreviations: *χ*², chi square; CFI, comparative fit index; CI, confidence interval of RMSEA; RMSEA, root mean square error of approximation; TLI, Tucker–Lewis index.

a Indicates the more constrained model to which this model is compared. Δ*χ* ^2^ *= *chi‐square difference. If chi‐square difference test is not significant, the constraints that fix parameters to be the same across groups are justified; if significant, the constraints are not justified and are not included in subsequent models.

**Table 3:** Coefficients of the unconstrained models to examine group differences in mean‐level change (Model 1A), rank‐order stability (Model 2A) and correlated change between self‐esteem and daily experiences (Model 3A)

Model		Job‐beginner group					Comparison Group				
		*β*	*B*	*SE*	*p*	95% CI	*β*	*B*	*SE*	*p*	95% CI
1A	Mean‐level change	.27	.13	.06	.022	0.04; 0.51	.13	.03	.03	.418	−0.19; 0.44
2A	Rank‐order change	**.52**	**.52**	**.11**	**.000**	**0.30; 0.75**	**.94**	**.84**	**.06**	**.000**	**0.72; 0.95**
3A	Correlated change										
	Satisfying achievement	**.42**	**.12**	**.04**	**.002**	**0.05; 0.20**	**.17**	**.02**	**.02**	**.284**	**−0.02; 0.06**
	Frustrating achievement	−.25	−.08	.04	.050	−0.15; 0.00	.02	.00	.02	.922	−0.04; 0.04
	Satisfying affiliation	.14	.05	.05	.282	−0.04; 0.14	.22	.03	.02	.191	−0.01; 0.07
	Frustrating affiliation	−.05	−.01	.02	.716	−0.05; 0.04	−.35	−.03	.01	.039	−0.06; −0.00

Note

*β* = Standardized coefficient estimates*. B* = Unstandardized coefficient estimates. Standard error (*SE*), *p* values and confidence intervals (CI) are shown for the unstandardized coefficients. Each line of the table presents the results for one multiple group model, respectively. Coefficients that differed at *p* < .05 across groups are in bold.

### 3.1 Group differences in mean‐level change (Aim 1)

Before estimating group differences, we obtained an estimate of the mean‐level change in self‐esteem for the whole sample. The model showed an excellent model fit to the data (*χ* ^2^ = 10.21, *df* = 12, CFI = 1.000; TLI = 1.003, RMSEA = .000, 90% CI = [0.000; 0.070]). The positive slope was significant and the effect size was small‐to‐medium (*B* = .07; *β* = .20, *SE* = .03, *p* = .032; CI = [0.01; 0.14]). On average, participants thus increased in their self‐esteem from T1 to T2.

To examine group differences in mean‐level changes in self‐esteem, we compared two nested models. We specified Model 1A, in which the slope was allowed to vary across groups, and compared it to a Model 1B, in which the slope was constrained to be equal across groups. As shown in Table 3 for Model 1A, self‐esteem significantly increased in the job‐beginner group (*β* = .27; *p* = .022), but the increase was nonsignificant in the comparison group (*β* = .13; *p* = .418). Both models showed an excellent model fit to the data (RMSEA < .05; CFI/TLI > .95; see Table 2). The model comparisons revealed a *p* value of .088 for the chi‐square difference test and the ΔCFI was .003. The former indicator suggests that there were no group differences in mean‐level change and the latter indicator suggests that there were group differences in mean‐level change. This provides weak evidence for group differences in mean‐level change suggesting that the mean‐level change in the job‐beginner group was somewhat, but not considerably larger than for the comparison group (for slopes, see Figure S1).

### 3.2 Group differences in rank‐order change (Aim 2)

We first obtained an estimate of the average rank‐order stability in self‐esteem for the whole sample by specifying an autoregressive path between T1 self‐esteem and T2 self‐esteem. The model showed an excellent model fit to the data (*χ* ^2^ = 10.21, *df* = 12, CFI = 1.000; TLI = 1.003, RMSEA = .000, 90% CI = [0.000; 0.070]) and the positive slope was *B* = .72; *β* = .75, *SE* = .06, *p* = .000; CI = [0.59; 0.84]. We next specified Model 2A, in which the autoregressive path was allowed to vary across groups, and compared it to a Model 2B, in which this path was constrained to be equal. Model 2A showed an excellent model fit (RMSEA < .05; CFI/TLI > .95) that was significantly better than the one for Model 2B (RMSEA > .08; CFI/TLI > .95; *χ* ^2^ difference test: *p* = .016; ΔCFI = 0.007; see Table 2). The coefficient for the autoregressive path between T1 and T2 self‐esteem was smaller in the job‐beginner group than in the comparison group (see Table 3 for coefficients). This finding indicated that the rank‐order stability was lower in the job‐beginner group than in the comparison group.

**Figure 1:**
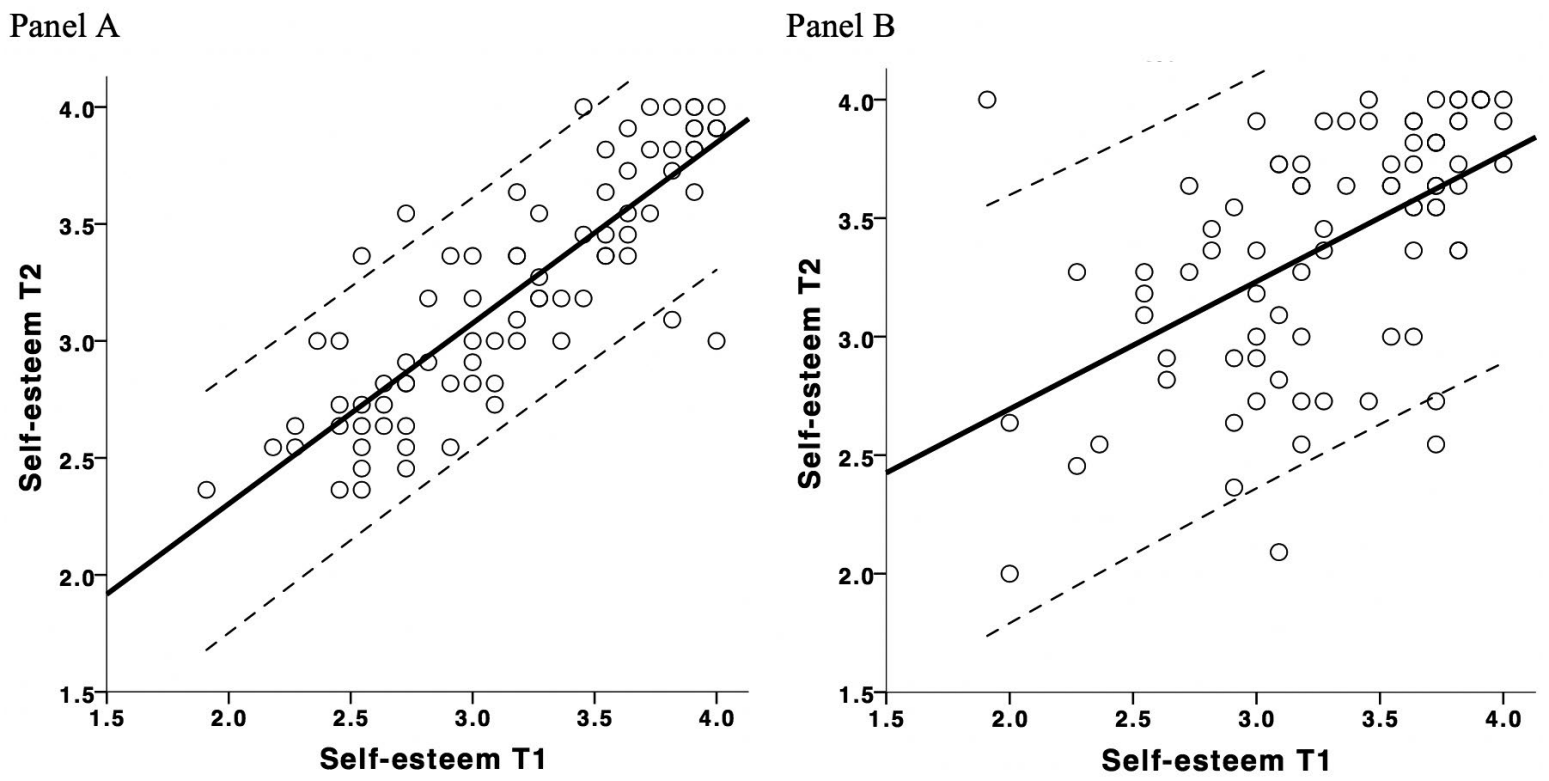
Raw self‐esteem data for the comparison group (Panel A) and the job‐beginner group (Panel B). The correlation between T1 and T2 self‐esteem is as depicted as the heavy black line and 95% confidence bounds of individuals are shown by dashed lines

For the job beginner group, there was a wider range of values than for the comparison group and an almost equal amount of increase and decrease in self‐esteem (see Supporting Information). Figure 1 illustrates the excess of retest instability in the job‐beginner group relative to the comparison group using manifest scores. As can be seen, the 95% confidence bounds of individuals were wider in the job‐beginner group than in the comparison group, which illustrates the job‐beginner's larger individual variability in self‐esteem change. If stability was perfect (if everyone had the exact same self‐esteem value at T1 and T2), the black line would be at 45 degrees (i.e., a diagonal). The line for the comparison group more resembles a diagonal than the line for the job beginners. The higher intercept for the job‐beginner group illustrates the slightly higher increase in self‐esteem.

### 3.3 Correlated change of self‐esteem and daily experiences (Aim 3)

Finally, we examined whether changes in self‐esteem were related to changes in the daily experience categories by specifying latent change score models. For each experience category, we specified one model in which the correlation between the slope of the experience variable and the slope of self‐esteem was allowed to vary across groups (unconstrained models, Models A) and one model in which it was constrained to be equal (constrained models, Models B).

We found one significant group difference. The model fit comparison for satisfying achievement‐related experiences shown in Table 2 indicated that the correlated change significantly differed across groups (Model 3.1 A vs. B; *χ* ^2^ difference test: *p* = .012; ΔCFI = .007). As shown in Table 3, change in satisfying achievement‐related experiences was significantly positively correlated with change in self‐esteem in the job‐beginner group (medium effect size), but they were unrelated in the comparison group (Figure S2 depicts the correlated change). There was a trend for frustrating achievement‐related experiences (Model 3.2 A vs. B; *χ* ^2^ difference test: *p* = .062; ΔCFI = .003), suggesting that the correlated change differed somewhat across groups. Change in frustrating achievement‐related experiences was negatively correlated with change in self‐esteem in the job‐beginner group (small effect size), but they were unrelated in the comparison group. The model fit comparisons for affiliation‐related experiences were not significant: satisfying affiliation‐related experiences (Model 3.3 A vs. B; *χ* ^2^ difference test: *p* = .680; ΔCFI = .000) and frustrating affiliation‐related experiences (Model 3.4 A vs. B; *χ* ^2^ difference test: *p* = .439; ΔCFI = .001).

In sum, the full‐time job and comparison groups differed in the correlated changes for achievement‐related experiences (which was significant for satisfying but not for frustrating experiences), whereas they did not differ in the correlated changes for affiliation. These findings suggested that for the job beginners (but not for the comparison group), rank‐order changes in satisfying and decrease in frustrating daily achievement‐related experiences were related to rank‐order changes in self‐esteem during the transition to work.

## 4 DISCUSSION

The aim of the present study was to examine self‐esteem change during a transition from university to work. We followed master's students from before to after their graduation, using a natural experiment by which half of them had started a full‐time job and the other half had not. At both waves, we assessed daily experiences. This study design went beyond most previous research as it allowed to systematically investigate how self‐esteem change in young adulthood relates to different transitional experiences and daily experiences.

The findings contribute several novel insights to the literature. First, we found weak evidence for group differences in mean‐level change, which suggests that the transition into full‐time work led to a slight increase in self‐esteem. Second, job beginners and the comparison group differed in their rank‐order stability: the job beginners had a lower rank‐order stability than the comparison group. Third, there were group differences in correlated change between achievement‐related experiences and self‐esteem (which was significant in the job‐beginner group but not in the comparison group), but there were no group differences in correlated change of affiliation‐related experiences and self‐esteem. The findings extend previous research in several ways, which will be discussed as follows.

### 4.1 Mean‐level change of self‐esteem in the transition to work

Our results suggested that starting a full‐time job after university graduation does not generally lead to a considerable boost in self‐esteem within the first year, but possibly to a small increase. Although the start of a full‐time job itself may be considered as a successful life path after finishing education from a developmental task perspective, the mere change in role status from studying to full‐time labor does not seem to be sufficient to considerably increase self‐esteem. The absence of a strong boost effect is in line with research on the college transition that suggests a drop during the initial phase of the transition but overall stability to slight positive trends across college (Chung et al., 2014; Shim et al., 2012; van der Velde et al., 1995). Our findings suggest that young adults are, similar to the into‐college experience, able to adapt to the out‐of‐college‐into‐job experience and thus maintain or even increase in their self‐esteem. Our finding however differs from the parenthood transition, which has been found to predict decrease in self‐esteem (Bleidorn, Arslan, et al., 2016). Perhaps this is due to the fact that the early phase of parenthood can be particularly stressful for which many parents are ill prepared, whereas the transition to work can be anticipated by traineeships and facilitated by on‐the‐job training.

Instead of considering job entry as binary indicator of whether a life transition is accomplished or not, it may be more promising to take a closer look at people's unique experiences during the transition. As we will discuss below, our findings suggest that the degree to which mastery experiences occur during the job transition is decisive for young adults’ self‐esteem change. This conclusion complements previous findings that indicated the sense of mastery in the peer domain to be the causal link between popularity and self‐esteem (Reitz, Motti‐Stefanidi, & Asendorpf, 2016). Although job beginners were faced with other types of daily experiences than the comparison group, the net valence of achievement‐related experiences did not differ across groups. This is in line with the notion that the job transition comes not only with positive experiences and opportunities to grow, but also with considerable challenges and experiences of failure (Schoon & Silbereisen, 2009). At the individual level, the varying degree of positive experiences during the job transition seems to be related to differential self‐esteem. Collectively, however, individuals’ upward and downward trajectories more or less cancel each other out (perhaps with a slight predominance in success experiences, as evidenced by the trend of a slightly increasing self‐esteem in the transition group). Hence, these findings suggest that although job entry can boost self‐esteem in individuals, many individuals experience negative or no change so the effect on the population is only minor.

### 4.2 Rank‐order stability of self‐esteem in the transition to work

The most compelling finding of our study was that individuals varied considerably in their self‐esteem change during the transition from university to work. The rank‐order stability in the job‐beginner group was significantly lower than in the comparison group. In the comparison group, the individuals’ self‐esteem levels before graduation was a better predictor of their self‐esteem level after graduation than for the job beginners: those with high levels of self‐esteem before graduation tended to have high levels of self‐esteem after, and vice versa. The magnitude of the rank‐order stability in the comparison group (*β* = .93) was in line with previous research (Kuster & Orth, 2013). The comparison group hence thus had a stable sense of self‐worth typical for their age group that was not destabilized by graduating from university.

The stability coefficients of the comparison group were however in stark contrast to the ones for the job‐beginner group (*β* = .52). Individuals who started a full‐time job followed different self‐esteem trajectories: some increased but many individuals also decreased or stayed stable. Those with high levels of self‐esteem before graduation thus did not necessarily have high levels of self‐esteem when in a full‐time job, and vice versa for low self‐esteem levels. This finding suggested that a transition to work can destabilize self‐esteem. It extends existing research on earlier life phases by demonstrating that self‐esteem can change in response to major life transitions in the middle of young adulthood. Replication studies are needed to corroborate this finding, but it provides first evidence suggesting that the transition from university to work has the potential to modify self‐esteem trajectories.

This destabilization finding corresponds to the notion in the life span literature that not all individuals follow the normative age trends, as some change to a larger degree, some do not change at all, and yet others change in ways that contradict general trends (Nesselroade, 1991; Reitz & Staudinger, 2017). In line with the notion that personality shows plasticity in response to environmental changes and demands, our findings suggested that the experience of the transition to work contributes to this individual variability in change in young adulthood. Our findings suggest that the unique environmental experiences that come with major life events and how well they are mastered can destabilize self‐esteem (Trzesniewski et al., 2003). Hence, life experiences seemed to contribute differentially to normative developmental changes in self‐esteem.

The destabilization finding has also wider‐reaching implications, as it sheds new light on theoretical approaches on self‐esteem development. Since the usually high rank‐order stability of self‐esteem seemed to decrease during job entry, self‐esteem may only be a highly stable characteristic as long as major life transitions are not considered. Our findings are thus in line with research highlighting that self‐esteem is characterized by both stable and more malleable parts (Donnellan et al., 2012). In addition, these findings demonstrated that the consideration of individual differences in self‐esteem change is crucial to understand the developmental processes underlying change (cf. Roberts & Mroczek, 2008). The mere focus on mean levels would have concealed that mean‐level stability resulted from the increase of some and the decrease of others, and thus, it would not have led to an exploration of the reasons for these individual differences.

### 4.3 The role of daily experiences in self‐esteem change

A compelling aspect of our study was that it extends beyond descriptive accounts of self‐esteem change during life transitions by examining whether self‐esteem change was linked to change in daily experiences. The findings make three major contributions to the literature. First, this study is one of the first to show that change in daily experiences is related to change in trait self‐esteem. The changes for those starting a full‐time job reflect the idiosyncratic nature of the experiences during the work transition in this sample. This finding provides supporting evidence for theoretical propositions that life transitions influence trait development not directly, but indirectly through altering social circumstances in everyday life (e.g., Hogan & Roberts, 2004). As indicated by the lower stability of daily satisfying achievement‐related experiences in the job‐beginner than in the comparison group (see Table 1: *r* = .47 vs. .60, respectively), the work transition seemed to have changed the job‐beginners' daily lives. Hence, the study provides a first indication that the change in daily life might be one explanation for the lower rank‐order consistency in self‐esteem for the job beginners.

Second, the pattern of findings that achievement‐related experiences were associated with self‐esteem change among job beginners but not the comparison group and that affiliation‐related experiences were unrelated to self‐esteem change provided a valuable first insight into the types of experiences that are relevant during the work transition. The most salient developmental task when starting to work is to succeed in work tasks and to learn new skills. Satisfying achievement‐related experiences are indicative for whether this task is accomplished successfully. Building on the notion that individuals’ self‐esteem is most contingent on domains in which they stake their self‐worth (Crocker & Wolfe, 2001), young adults who transition into work life seem to stake their self‐worth on succeeding in the work domain. As a result, it seems that the degree to which job beginners succeeded in their work‐related tasks in everyday life is one reason for their destabilization of self‐esteem.

Affiliation experiences, in contrast, appeared less relevant for job beginners, perhaps because they are less informative about the success in this salient task. This interpretation was supported by the finding that job beginners experienced less affiliation experiences over time and significantly fewer satisfying affiliation experiences at T2 than the comparison group, which might be due to time constraints of their full‐time job. Interestingly, this did not affect self‐esteem negatively. Affiliation‐related experiences have however been found to impact self‐esteem in previous studies, but most of them examined adolescents (e.g., peer popularity in the school context; Reitz et al., 2016)—a developmental phase in which the need to affiliate is highly salient. It would be an interesting line of future research to examine whether those experiences that indicate the mastery of the most salient task of the respective developmental period have the strongest effects on self‐esteem. Whereas achievement‐related experiences seem to be most relevant in the transition to work, the focus may shift during other life transitions. More research that directly compares success and failure across a broader range of success indicators and developmental periods is needed to further explore these possibilities.

Third, on a broader level, the finding also provided novel insights for the advancement of theories on self‐esteem and its development. The current debate of whether self‐esteem is dominated by agentic versus communal information may be refined by a more developmentally sensitive perspective that accounts for the importance of both types of experiences. In line with the notion of the two‐factor approach of self‐esteem (Mruk, 2013), we contend that self‐esteem is both based on agentic and communal information, but their relative importance might vary across developmental periods that differ in the salience of communal and agentic demands. In other words, the contingencies of self‐esteem may change across developmental periods and transitions. Self‐esteem could thus be considered as an indicator of developmental success: successful mastery of the transitional challenges may convey a sense of accomplishment and hence, impact self‐esteem.

### 4.4 Limitations and future directions

A number of study strengths allowed us to provide novel insights into the development of self‐esteem. We assessed young adults before and after starting a full‐time job and compared their self‐esteem stability and change to a comparable group that did not (yet) experience this transition. This quasi‐experimental design allowed to examine the impact of a transition from university to full‐time work. The daily diary measurements of experiences allowed us to assess the individuals’ typical daily experiences before and after the transition while avoiding biases of retrospective assessments. The achievement‐ and affiliation‐based experiences provided initial evidence for which kinds of daily experiences may help explain differential self‐esteem change in the transition from university to work. Despite these strengths, some limitations need to be considered which provide avenues for future research.

First, future studies with larger sample sizes are needed to replicate our findings. Given that we found a significant mean‐level increase in self‐esteem in the job‐beginner group with a medium effect size, no mean‐level change in the comparison group, but only small group differences, replication studies with larger sample sizes might find significant group differences in mean‐level change. Larger sample sizes would also allow to compare subgroups to examine if their daily lives, role‐related demands, and career goals differ and, as a result, their self‐esteem trajectories. The daily lives of those in full‐time jobs might be more different from those who are unemployed than from those in part‐time jobs and internships and hence, one might be more likely to find group differences in mean‐level change for full‐time versus unemployed than for full‐time versus part‐time. Unemployed might however not be the ideal comparison group if one is interested in examining the impact of the work transition, as their self‐esteem stability may decrease if they are not able to find a job for longer periods (Galambos, Barker, & Krahn, 2006). Ideally, one would also include a comparison group with more stable environments, such as students who follow a post‐master education, to disentangle maturation and transition effects.

Second, we only measured self‐esteem twice. An important advance for future research would be to have more assessments to assess the shape of the self‐esteem trajectory more precisely (e.g., to cover nonlinear trends, as when self‐esteem decreases right after the job transition but then rebounds) and to examine anticipatory changes (Denissen, Luhmann, Chung, & Bleidorn, 2019). Additional long‐term assessments will also allow to examine if there are differences in mean‐level change that only appear after more than a year and if and when the decreased rank‐order stability among job beginners increases again. Moreover, future research should examine the extent to which some of the individual variability in self‐esteem that we observed are due in part to individual differences in state reactivity (cf. Kernis, 2003). Daily assessments of state self‐esteem can also be linked to daily assessments of events to examine their interplay and to illuminate the underlying mechanisms, such as whether changes in daily experiences accumulated and manifested in trait self‐esteem (see Borghuis et al., 2018).

Third, like any study based on comparisons of non‐randomized groups, our findings cannot be considered to be definitive. We encourage other researchers to carry out comparable analyses to examine the generalizability of our findings, as our sample is not representative of all university‐to‐work transitions in Germany or comparable cultures. Moreover, an interesting extension of our research would be to focus on non‐WEIRD samples since developmental tasks and self‐esteem trajectories can be culture‐specific (Bleidorn, Buyukcan‐Tetik, et al., 2016).

## 5 CONCLUSION

The present study extended prior research on self‐esteem development in young adulthood by suggesting that the transition from university to full‐time employment is an important context for self‐esteem development. The results provided initial evidence that the transition from university to work can destabilize self‐esteem as indicated by a decrease in rank‐order stability. This destabilization pattern sheds new light on an important topic in the field of self‐esteem development: it suggests that the high stability of self‐esteem usually found in the literature might not hold during a major life transition. Accounting for daily life experiences allowed us to gain first insights into the processes leading to this destabilization pattern: the changes in daily satisfying achievement‐related experiences during the university‐to‐work transition were related to changes in self‐esteem. As satisfying achievement‐related experiences indicate the degree to which job beginners master the work transition, we speculate that developmentally salient daily experiences during life transition might help understand self‐esteem development in young adulthood. Future studies should apply an individualized and developmental approach that accounts for the uniqueness of individuals’ major life transitions to better understand self‐esteem development in young adulthood.

## CONFLICT OF INTEREST

The authors declared no potential conflicts of interest with respect to the research, authorship, and/or publication of this article.
