# Supplementary material for: Self‐esteem change during the transition from university to work
Source: J Pers. 2019 Oct 17;88(4):689–702. doi: 10.1111/jopy.12519 (PMC7383858; doi:10.1111/jopy.12519)
Supplement: Supplementary file 1 [file JOPY-88-689-s001.docx]

**Supplemental Materials**

**Self-Esteem Change During the Transition from University to Work**

**Comparisons of Job Beginner Group and Comparison Group at Time 1**

We examined whether the groups were comparable at T1 in a number of characteristics. We conducted a series of t-tests and found no group differences at T1 in the study variables (self-esteem, the events), in demographic characteristics (age, gender distribution), or in other potentially relevant variables that were part of the larger longitudinal study (which was concerned with changes in a range of personality and well-being indicators over time, such as, for example, motive dispositions, the Big Five, life satisfaction or depression; see Table S1). As the groups did not differ on any of these variables, they can be considered comparable before the job transition. These findings suggest that the study design qualifies as a natural experiment, as it is very unlikely that group differences in self-esteem change in the following analyses are due to other differences than experiencing job entry.

**Comparisons of the Transitions of the Job Beginner and Part-Time/Internship Groups**

To get a better understanding of the circumstances of the two groups, which will also contribute to circumvent the generalizability of our findings, we compared the two groups on several transition-related characteristics (see Table S2). We compared the job experience for the full-time job group and those in the comparison group who were working in part-time jobs or internships (*n*=59). There was no group difference in the response to the question “Did you feel like you had a choice while choosing a job?”. Of the part-time group (the comparison group minus the unemployed), 64% said they had not much choice and of the full-time job group 61% said they had not much choice. There was no significant group difference in the number of job offers received, nor in the number of applications written (but there was a trend that the full-time job group had on average more applications written than the comparison group). These characteristics suggest that the two groups did not differ significantly in the level of voluntariness of their group membership.

The job-beginner group started with their jobs *M*=6.78 months (*SD*=2.73) prior to T2 and those in the comparison group who had part-time jobs or internships started with their jobs 5.86 months prior to T2 (*SD*=3; but they did not significantly differ in their starting dates). As to be expected, the full-time job group indicated to have significantly higher income levels and higher challenging job demands than those in part-time jobs or internships. In sum, these group comparisons demonstrate that the only difference between the job beginners and the comparison group is that the former has already made the transition into a full-time job and is hence on a career path, which can be considered as successful mastery of the job transition. In contrast, those with part-time jobs and internships had more casual jobs and hence have not yet reached the developmental milestone. Hence, the formation of the job-beginner group and the comparison group was justified.

**Subgroup Comparisons**

The casually working group (part-time and internship, *n*=59) resembled the unemployed group (*n*=26) in several characteristics (although all following comparisons should be interpreted with power considerations in mind). We found that they did not differ in any demographic or study variable at T1 at a significance level of .05, which points to their comparability before the transition. They did not differ in their frequency of experiencing any of the event categories at T2 (means and SDs for unemployed/part-time and internship: achievement satisfaction: 2.70(.60)/2.96(.61); achievement frustration: 2.70(.74)/2.55(.70); affiliation satisfaction: 3.74(.66)/3.45(.68); affiliation frustration: 1.95(.44)/2.03(.43)). The casually working group and the unemployed did neither differ in their mean-level changes (i.e., both groups showed non-significant change: *F*(1, 83)=.087, *p*=.769; mean increase and SD for unemployed/ part-time and internship: .05(.34)/.03(.26)) nor in their rank-order changes in self-esteem (i.e., both groups showed similar levels of rank-order stability that were larger than for the job beginners; full-time/part-time and internship/unemployed: achievement satisfaction: .47/.65/.56; achievement frustration: .49/.61/.70; affiliation satisfaction: .45/.54/.49; affiliation frustration: .45/.48/.46). Together, these analyses justify subsuming the casual job-beginner group and unemployed in the comparison group.

**Cronbach’s Alphas for the daily experiences for both groups**

Cronbach’s alphas for the daily experiences for the job beginners/comparison groups were: satisfying achievement experiences at T1: .69/.76; T2: .63/.65; frustrating achievement experiences at T1: .84/.72; at T2: .63/.78; satisfying affiliation experiences at T1: .84/.75; at T2: .87/.84; frustrating affiliation experiences at T1: .01/.21; at T2: .34/.40

**Measurement Invariance**

We tested four levels of factorial invariance, each imposing more constraints. If the group constraints were justified, indicated by a non-significant decrease in fit, they were retained in the subsequent models. Results are documented in Table S3 and show that even the most restrictive models still had good model fit. These results indicated that the self-esteem construct had the same meaning and structure at both time points and both groups. Hence, mean-level and rank-order change and group differences therein can be meaningfully interpreted. All analyses use a time- and group-invariant measurement model.

**Measurement invariance across time.** Testing for measurement invariance across time ensures that changes are due to real changes in the underlying construct and not to variance in trait measurement over time (Bollen & Curran, 2006). We allowed the indicator residuals to correlate across time to account for the effects of the specific indicators other than those of the underlying factors (Bollen & Curran, 2006). We set the factor loading of the first indicator to 1 at all time points, allowing the loadings of the other indicators to vary. We tested four levels of factorial invariance, each imposing more constraints. If the constraints were justified they were retained in the subsequent models.

First, we tested for configural invariance by constraining the pattern of factor loadings to be equal across time. The three item parcels were allowed to load on a latent self-esteem variable at each measurement point. Second, we tested for weak factorial invariance by constraining the factor loadings to be equal across time. Third, we tested for strong (or metric) factorial invariance by constraining factor loadings and intercepts to be equal across time. Finally, we tested for strict measurement invariance by constraining residual variances to be equal over time. None of the time-constraints resulted in a worsening of the model fit. Analyses showed that even the most restrictive model still had good model fit (CFI > .90, RMSEA < .08, no decline in CFI > .02). All subsequent models were therefore based on this more parsimonious measurement model with time-invariant factor loadings, indicator intercepts and error variances.

**Measurement invariance across groups.** Next, we tested for measurement invariance across the job versus comparison groups to ensure that group differences are due to real differences in the underlying construct and not to variance in the factor structure across groups. We included the multiple group models into the strict time-invariant measurement model described above and followed the same procedure. We tested for configural, weak and strong invariance for both measurement points. As can be seen in Table S3, strong factorial invariance held across groups. All subsequent analyses were therefore based on a measurement model of strong factorial invariance across groups.

**Magnitude of Individual Variation**

To assess the magnitude of variation, we followed the recommendation of Raudenbush and Bryk (2002, p. 78) and calculated the 95% plausible value range (PVR) of the slopes for both groups using the information of the average slope and the slope standard deviation. The slope parameters for self-esteem ranged between -0.36 and 0.44 for 95% of the participants in the comparison group and it ranged between -0.87 and 1.07 for 95% of the participants in the job-beginner group. The 95% PVR illustrates not only that there was a wider range of plausible values for the job-beginner group than for the comparison group, but also that there was an almost equal amount of increase and decrease in self-esteem.

Table S1

*Results of t-test and Descriptive Statistics for Variables at T1 by Group*

|  | Comparison Group | | |  | Job Beginner Group | | |  |  |  |
| --- | --- | --- | --- | --- | --- | --- | --- | --- | --- | --- |
|  | *M* | *SD* | *n* |  | *M* | *SD* | *n* | *t* | *df* | *p* |
| Self-esteem (1-5) | 3.17 | 0.55 | 85 |  | 3.29 | 0.51 | 78 | -1.46 | 161 | .145 |
| Achievement, satisfying events (1-5) | 2.79 | 0.64 | 85 |  | 2.82 | 0.56 | 78 | -.35 | 161 | .723 |
| Achievement, frustrating events (1-5) | 2.84 | 0.64 | 85 |  | 2.85 | 0.60 | 78 | -.06 | 161 | .951 |
| Affiliation, satisfying events (1-5) | 3.39 | 0.59 | 85 |  | 3.46 | 0.66 | 78 | -.76 | 161 | .445 |
| Affiliation, frustrating events (1-5) | 2.08 | 0.38 | 85 |  | 1.98 | 0.32 | 78 | -.76 | 161 | .445 |
| Sex | .29 | 0.46 | 85 |  | .32 | 0.47 | 78 | -.36 | 161 | .717 |
| Age | .32 | 0.47 | 78 |  | 26.87 | 2.57 | 78 | .89 | 161 | .372 |
| Life Satisfaction (The Satisfaction with Life Scale) (1-5) | 4.13 | 1.00 | 85 |  | 4.22 | 0.92 | 78 | -.62 | 161 | .537 |
| Depression (Beck-Depression-Inventar, Schmitt & Maes, 2000) (1-6) | 2.57 | 0.72 | 85 |  | 2.45 | 0.65 | 78 | 1.11 | 161 | .269 |
| Verbal Intelligence (Mehrfach-Wortschatz-Intelligenztest, MWT_B) (0-37) | 25.25 | 3.65 | 85 |  | 24.72 | 3.55 | 76 | .92 | 159 | .359 |
| Knowledge economy (Der Wilde-Intelligenztest, WIT-2) | 17.19 | 2.12 | 85 |  | 17.72 | 1.92 | 76 | -1.67 | 159 | .096 |
| Knowledge IT (Der Wilde-Intelligenztest, WIT-2) | 16.07 | 3.18 | 85 |  | 16.24 | 3.09 | 76 | -.34 | 159 | .738 |
| D2 Test of Attention | 4.38 | 1.97 | 84 |  | 4.63 | 2.16 | 74 | -.75 | 156 | .454 |

Table S2

*Results of t-test and Descriptive Statistics for Job Transition-Relevant Variables at T2 by Group*

|  | Comparison Group | | |  | Job Beginner Group | | |  |  |  |
| --- | --- | --- | --- | --- | --- | --- | --- | --- | --- | --- |
|  | *M* | *SD* | *n* |  | *M* | *SD* | *n* | *t* | *df* | *p* |
| How many applications did you write? | 15.00 | 21.25 | 58 |  | 21.48 | 33.77 | 77 | -1.28 | 133 | .202 |
| How many job offers have you received? | 2.95 | 3.79 | 59 |  | 2.41 | 1.56 | 78 | 1.14 | 135 | .258 |
| Did you feel like you had a choice while choosing your job? (1=not much, 2 = much) | 2.41 | 1.56 | 78 |  | 1.38 | 0.49 | 78 | -.34 | 135 | .733 |
| Final grade | 2,02 | 1.97 | 83 |  | 1.98 | 1.36 | 77 | .15 | 158 | .881 |
| When did you start with your current job? (1-9 months prior) | 5.86 | 3.14 | 59 |  | 6.78 | 2.73 | 78 | -1.79 | 115 | .076 |
| Self-reported income group (1=less than 1000 p. m., 7= more than 6000 p. m.) | 1.61 | 0.95 | 59 |  | 2.65 | 0.91 | 78 | -6.53 | 135 | .000 |

Table S3

*Fit Indices for Testing Measurement Invariance for Self-Esteem Across Time and Group*

|  | Model | *χ^2^* | *df* | CFI | TLI | RMSEA | 90% CI | Δ*χ^2^* | Δ*df* | *p* |
| --- | --- | --- | --- | --- | --- | --- | --- | --- | --- | --- |
| Time | Configural | 2.38 | 5 | 1.000 | 1.012 | .000 | [.000, .070] |  |  |  |
|  | Weak | 3.46 | 7 | 1.000 | 1.011 | .000 | [.000, .056] | 1.08 | 2 | .582 |
|  | Strong | 4.67 | 9 | 1.000 | 1.011 | .000 | [.000, .047] | 1.21 | 2 | .547 |
|  | Strict | 10.21 | 12 | 1.000 | 1.003 | .000 | [.000, .070] | 5.54 | 3 | .136 |
| Group | Configural | 30.75 | 24 | 0.990 | 0.988 | .059 | [.000, .113] |  |  |  |
|  | Weak | 31.82 | 26 | 0.992 | 0.990 | .052 | [.000, .107] | 1.07 | 2 | .587 |
|  | Strong | 34.09 | 29 | 0.993 | 0.992 | .046 | [.000, .100] | 2.27 | 3 | .518 |

*Note.* χ² = Chi square; CFI = Comparative fit index; TLI = Tucker–Lewis index; RMSEA = Root mean square error of approximation; CI = confidence interval of RMSEA. ΔCFI = Difference in CFI from previous model. If CFI decreases > .001 when compared to more constrained model, the constraints that fix parameters to be the same across time or group are justified and retained in subsequent models.

*Figure S1.* Mean-level change based on manifest variables for the job beginner group and the comparison group on the 1-5 self-esteem scale. Error bars are 95% confidence intervals.

Panel A Panel B


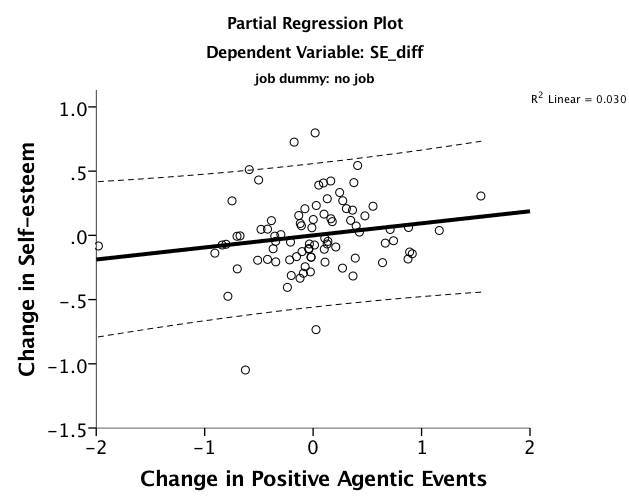

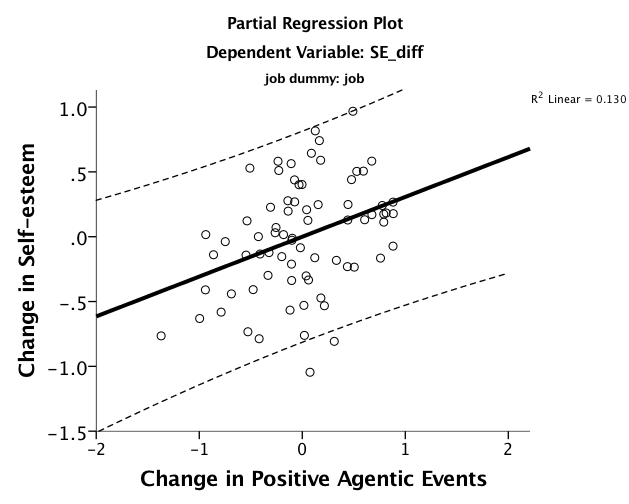


*Figure S2.* Regression plot that shows the association of change in self-esteem and change in satisfying achievement (agentic) events for the comparison group (Panel A) and job beginners (Panel B) while accounting for change in frustrating achievement events and satisfying and frustrating affiliation events.
